# NO_2_ sensing properties of WO_3_-decorated In_2_O_3_ nanorods and In_2_O_3_-decorated WO_3_ nanorods

**DOI:** 10.1186/s40580-019-0205-2

**Published:** 2019-12-13

**Authors:** Bumhee Nam, Tae-Kyoung Ko, Soong-Keun Hyun, Chongmu Lee

**Affiliations:** Department of Materials Science and Engineering, Inha University, 253 Yonghyun-dong, Nam-gu, Incheon, 402-751 Republic of Korea

**Keywords:** Gas sensor, Heterostructure, WO_3_, In_2_O_3_, NO_2_

## Abstract

In_2_O_3_ nanoparticle (NP)-decorated WO_3_ nanorods (NRs) were prepared using sol–gel and hydrothermal methods. The In_2_O_3_ NRs and WO_3_ NPs were crystalline. WO_3_ NP-decorated In_2_O_3_ NRs were also prepared using thermal evaporation and hydrothermal methods. The NO_2_ sensing performance of the In_2_O_3_ NP-decorated WO_3_ NR sensor toward NO_2_ was compared to that of the WO_3_ NP-decorated In_2_O_3_ NR sensor. The former showed a high response to NO_2_ due to a significant reduction of the conduction channel width upon exposure to NO_2_. In contrast, the latter showed a far less pronounced response due to limited reduction of the conduction channel width upon exposure to NO_2_. When the sensors were exposed to a reducing gas instead of an oxidizing gas (NO_2_), the situation was reversed, i.e., the WO_3_ NP-decorated In_2_O_3_ NR exhibited a stronger response to the reducing gas than the In_2_O_3_ NP-decorated WO_3_ NR sensor. Thus, a semiconducting metal oxide (SMO) with a smaller work function must be used as the decorating material in decorated heterostructured SMO sensors for detection of oxidizing gases. The In_2_O_3_ NP-decorated WO_3_ NR sensor showed higher selectivity for NO_2_ compared to other gases, including reducing gases and other oxidizing gases, as well as showed high sensitivity to NO_2_.

## Introduction

Despite the numerous merits of semiconducting metal oxides (SMOs) as sensor materials there are still certain limitations, such as their relatively low response to gases at room temperature and dissatisfactory selectivity [1]. To address the dissatisfactory sensing properties, various strategies have been attempted, including noble metal catalyst doping, heterojunction formation, and radiation-assisted treatment with energetic particles including ion beams, electrons, and ultraviolet (UV) lights [2–4]. Of these techniques, heterostructure formation is plausibly most widely studied and is used for the fabrication of chemiresistive nanostructured gas sensors. There are several types of heterostructures including p-n, n–n and p–p heterostructures. Generally, p–p heterostructures are less commonly utilized because of their inferior sensing properties, whereas n–n heterostructures are as widely utilized as the p–n counterparts because of their superior sensing properties [5]. However, strangely, n–n heterostructures have not been studied as intensively as the p–n congeners. The enhanced sensing properties of n–n heterostructures are mainly due to the resistance modulation at the n–n heterojunctions in n–n heterostructures. Various heterostructure combinations are known, such as a simple mixture of two different types of n-SMOs [6], bi-layer type n–n nanostructures [7], n–n core–shell structures [8], a single type of n-SMO nanostructure decorated with another type of n-SMO nanoparticles (NPs) [9], etc.

This study focuses on, decorated n–n heterostructures. WO_3_ and In_2_O_3_ are chosen as sensor materials for detecting a typical oxidizing gas, NO_2_. The sensing properties of In_2_O_3_ NP-decorated WO_3_ nanorods (NRs), WO_3_ NP-decorated In_2_O_3_ NRs, pristine WO_3_ NRs, and pristine In_2_O_3_ NRs are compared and the differences in the sensing properties of these four nanostructures are analyzed and the origin of the differences is discussed in detail.

## Methods

### Preparation of In_2_O_3_ nanoparticles-decorated WO_3_ nanorods

High purity In_2_O_3_ NPs were synthesized using a sol–gel method [10]. Indium acetate ([In(C_2_H_3_O_2_)2·H_2_O]; 0.6695 g) was dissolved in diethylene glycol and stirred for 5 min. The solution was homogenized by heating to 130 °C and 3 mL of 3 *N*-nitric acid was added to the solution and stirred well. The solution was heated at 180 °C for 5 h, and pure yellowish In_2_O_3_ NPs were precipitated. The In_2_O_3_ NPs were dried at 400 °C for 2 h and then calcined at 500 °C for 1 h to obtain the pure In_2_O_3_ NPs. The WO_3_ NRs were synthesized by using a low-temperature hydrothermal method [11]. Sodium tungstate (1.956 mL) and oxalic acid (1.512 mL) were dissolved in distilled water (50 mL). The solution was acidified to PH 0.7–0.9 by mixing with 3 mol/L HCl solution. A transparent precursor solution was formed and 3 g of K_2_SO_4_ was added to the solution. The mixed solution was maintained in an autoclave at 100 °C for 24 h, cooled to room temperature, and was centrifuged to collect the green product. The product was rinsed with ethanol and dried at 60 °C for 1 h to obtain pure WO_3_ NRs. The substrate on which the WO_3_ NRs were synthesized was placed on a spin coater and then rotated at 500 rpm. The In_2_O_3_ NPs synthesized via the sol–gel method were dispersed in ethanol with a micropipette and the ethanolic dispersion of In_2_O_3_ NPs was dropped on the rotating WO_3_ NR substrate.

### Preparation of WO_3_ nanoparticles-decorated In_2_O_3_ nanorods

In_2_O_3_ NRs were synthesized using a thermal evaporation method [12]. A 3 mm thick gold film—coated p-type Si (100) substrate was placed on the top of an alumina boat containing a mixture of In_2_O_3_ powders and positioned at the center of a horizontal quartz tube furnace. The furnace was heated to 900 °C and maintained at that temperature for 30 min under argon gas at a constant flow rate of 200 cm^3^/min. The WO_3_ NPs were synthesized using a hydrothermal method [13]. WO_3_ powders (2 mL) were dissolved in 48 mL of hydrochloric acid in sonicater. The pH of the solution was controlled at 7 using sodium hydroxide. After sonication of the solution for 6 h the precipitated powders were collected by removing the liquid, leaving the powders behind. The powders were placed into a hydrothermal synthesizer containing ethanol and the synthesizer was placed in an oven and heated at 180 °C for 12 h. WO_3_ NPs were synthesized in the hydrothermal synthesizer. The substrate on which the In_2_O_3_ NRs were synthesized by the thermal evaporation method was placed in a beaker containing ethanol and then ultrasonicated to separate the In_2_O_3_ NRs from the substrate. Meanwhile, the WO_3_ NPs synthesized by the hydrothermal method were dispersed in ethanol. The two solutions (In_2_O_3_ NRs dispersed in ethanol and the WO_3_ NPs dispersed in ethanol) were mixed and the mixed solution was exposed to UV (254 nm) irradiation for 12 h using a UV lamp. The mixed solution was then annealed under argon atmosphere at 400 °C for 1 h in an annealing furnace.

### Fabrication of chemiresistive sensors

The In_2_O_3_ NP-decorated WO_3_ NRs and WO_3_ NP-decorated In_2_O_3_ NRs grown on the Si substrate were dispersed ultrasonically in isopropyl alcohol. A multiple-networked chemiresistive sensor was fabricated by pouring the solution containing the precursors of the two different nanostructures onto SiO_2_/Si substrates with a patterned interdigital electrode with a double layer comprising separate layers of Ti (10 nm) and Au (100 nm): the assembly was dried at 150 °C for 1 min. For comparison of the sensing properties, pristine In_2_O_3_ and WO_3_ NR sensors were also fabricated in a similar manner.

### Characterization

The microstructures and phases of the synthesized NR samples were examined by scanning electron microscopy (SEM) and X-ray diffraction (XRD), respectively. The microstructures and phases of the samples were examined further by transmission electron microscopy (TEM).

### Gas sensing tests

The NO_2_ sensing performances of the fabricated sensors were examined using a custom-made gas sensing system. The concentration of NO_2_ gas was controlled precisely in the concentration range of 5–200 ppm by mixing NO_2_ with dry synthetic air using the mass flow controllers. Electrical measurements to examine the sensing properties of the sensors were conducted at room temperature under 50% relative humidity. The detailed sensing test procedure is described elsewhere [14]. The response of the sensors to NO_2_ was evaluated by using the *R*_*g*_*/R*_*a*_ ratio, where *R*_*g*_ and *R*_*a*_ are the resistances of the sensor measured in the presence of air and NO_2_, respectively. The response and recovery times were determined by measuring the times required to reach 90% of the total change in the resistance of the sensor after exposure of the sensor to the analyte gas and ambient air, respectively.

## Results and discussion

**Fig. 1:**
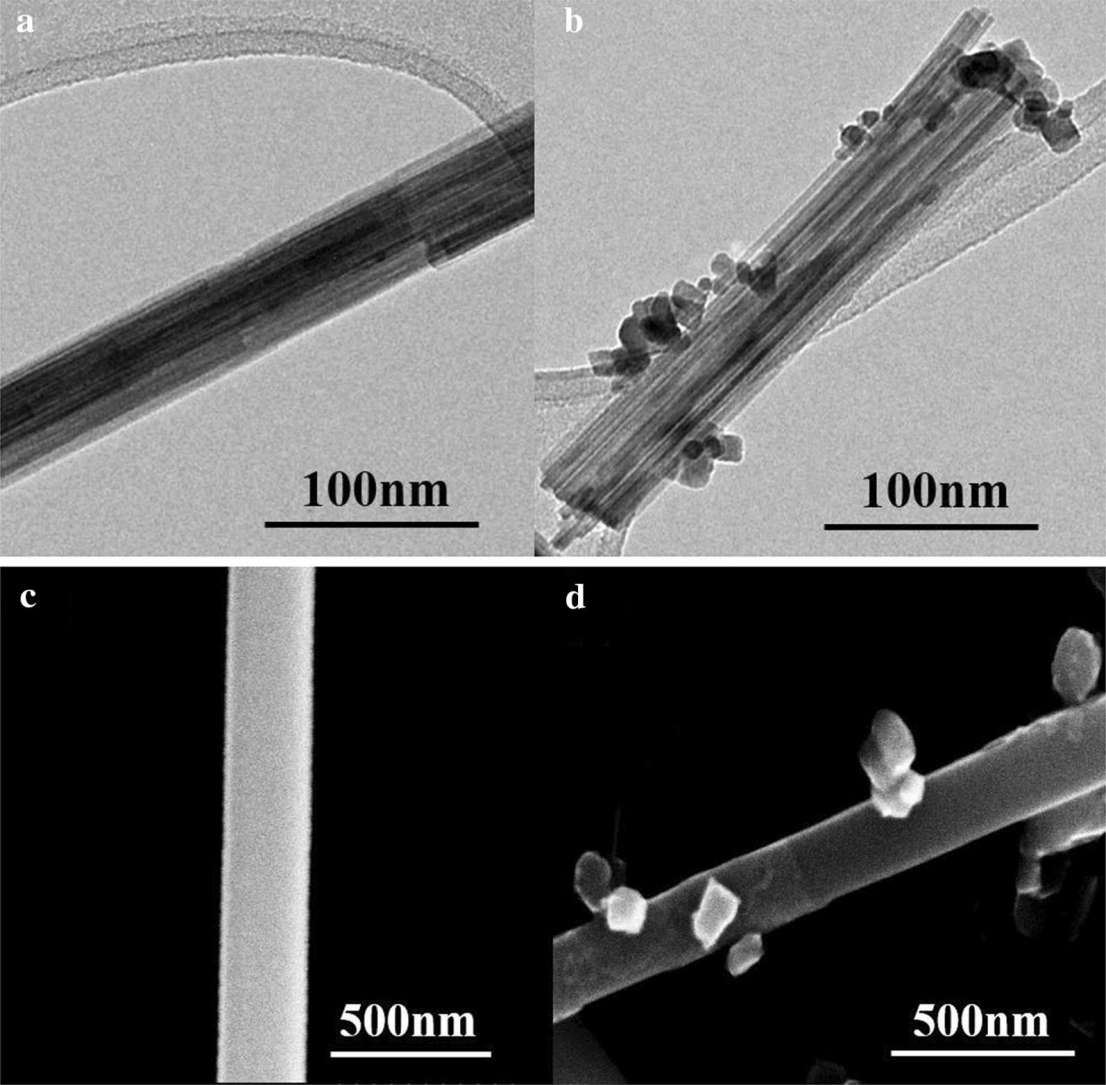
Low-magnification TEM images: **a** pristine and **b** In_2_O_3_ NPs-decorated WO_3_ NRs and SEM images: **c** pristine and **d** WO_3_ NPs-decorated In_2_O_3_ NRs

Figure 1a, b show low-magnification TEM images of the pristine and In_2_O_3_ NPs-decorated WO_3_ NRs, respectively. The average diameter of the WO_3_ NRs was ~ 50 nm and the length of the WO_3_ NRs ranged from 200 to 1100 nm. The average diameter of the In_2_O_3_ NPs on the WO_3_ NRs was 20 nm. The SEM images of the pristine and WO_3_ NP-decorated In_2_O_3_ NRs are exhibited in Fig. 1c, d. The average diameter of the In_2_O_3_ NRs was 250 nm and the lengths of the In_2_O_3_ NRs ranged from 1 to 10 μm. The average diameter of the WO_3_ NPs on the In_2_O_3_ NRs was 140 nm. Hence, the average diameter of the WO_3_ NPs on the In_2_O_3_ NRs was ~ 7 times larger than that of the In_2_O_3_ NPs on the WO_3_ NRs. The difference in size might be due to the different preparation methods (sol–gel versus hydrothermal methods).

**Fig. 2:**
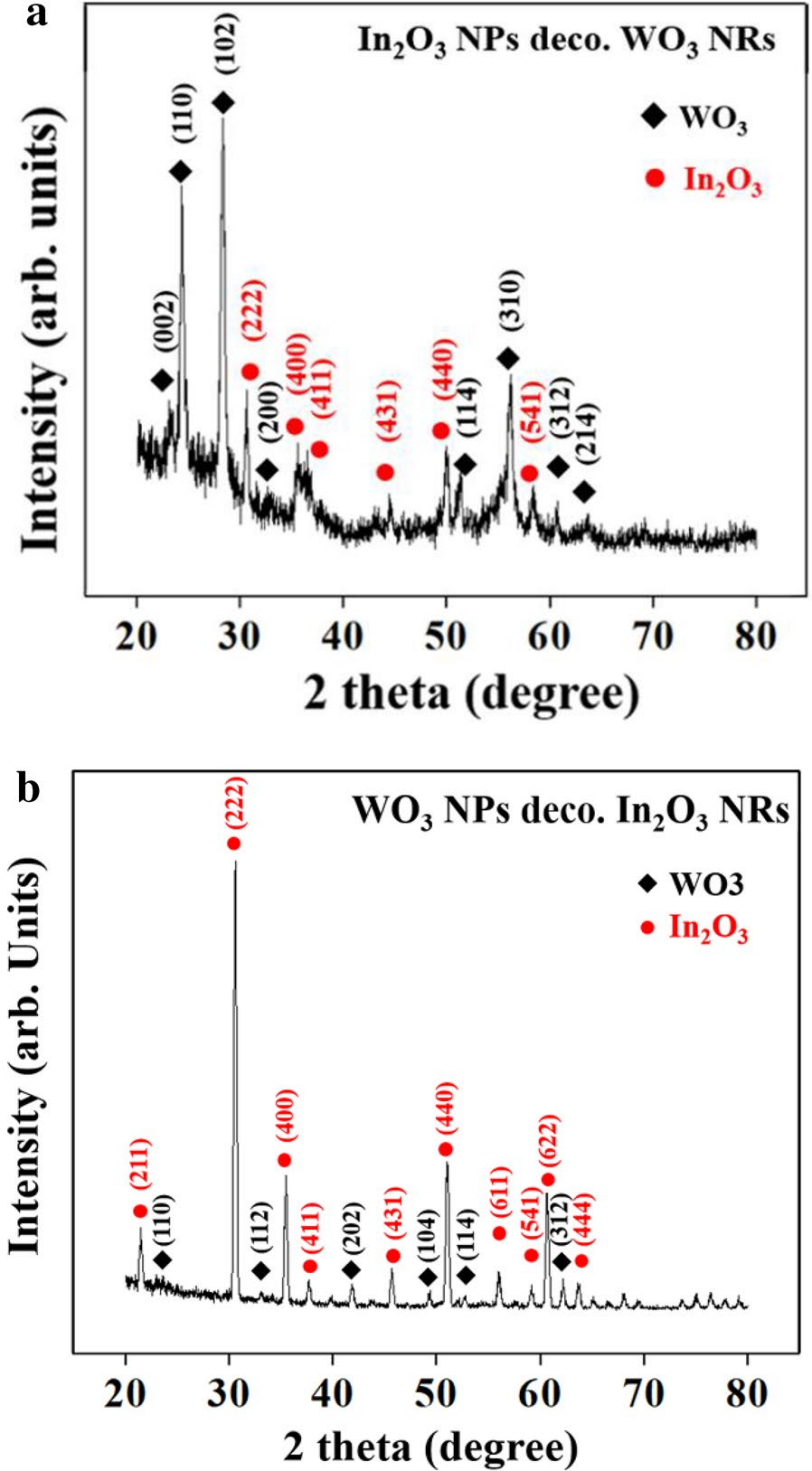
XRD patterns of **a** In_2_O_3_ NPs-decorated WO_3_ NRs and **b** WO_3_ NPs-decorated In_2_O_3_ NRs

Figure 2a, b show the XRD patterns of the In_2_O_3_ NP-decorated WO_3_ NRs and WO_3_ NP-decorated In_2_O_3_ NRs, respectively. In the former pattern, the WO_3_ NRs exhibited relatively sharp and intense reflection peaks, assigned to the primitive tetragonal structured WO_3_ (JCPDS card No. 89-4481, a = 0.5275 nm, c = 0.7846 nm). In contrast, the In_2_O_3_ NPs exhibited relatively less sharp and less intense reflection peaks, assigned to body-centered cubic In_2_O_3_ with a lattice constant of *a* = 1.011 nm (JCPDS No. 89-4595). The lower intensity peaks for In_2_O_3_ compared to WO_3_ might be due to the smaller volume of the In_2_O_3_ NPs relative to that of the WO_3_ NRs. In contrast, in the latter pattern (Fig. 2b), In_2_O_3_ peaks were taller and sharper than WO_3_ peaks, which might be due to the larger volume of In_2_O_3_ NRs than those of the WO_3_ NPs.

**Fig. 3:**
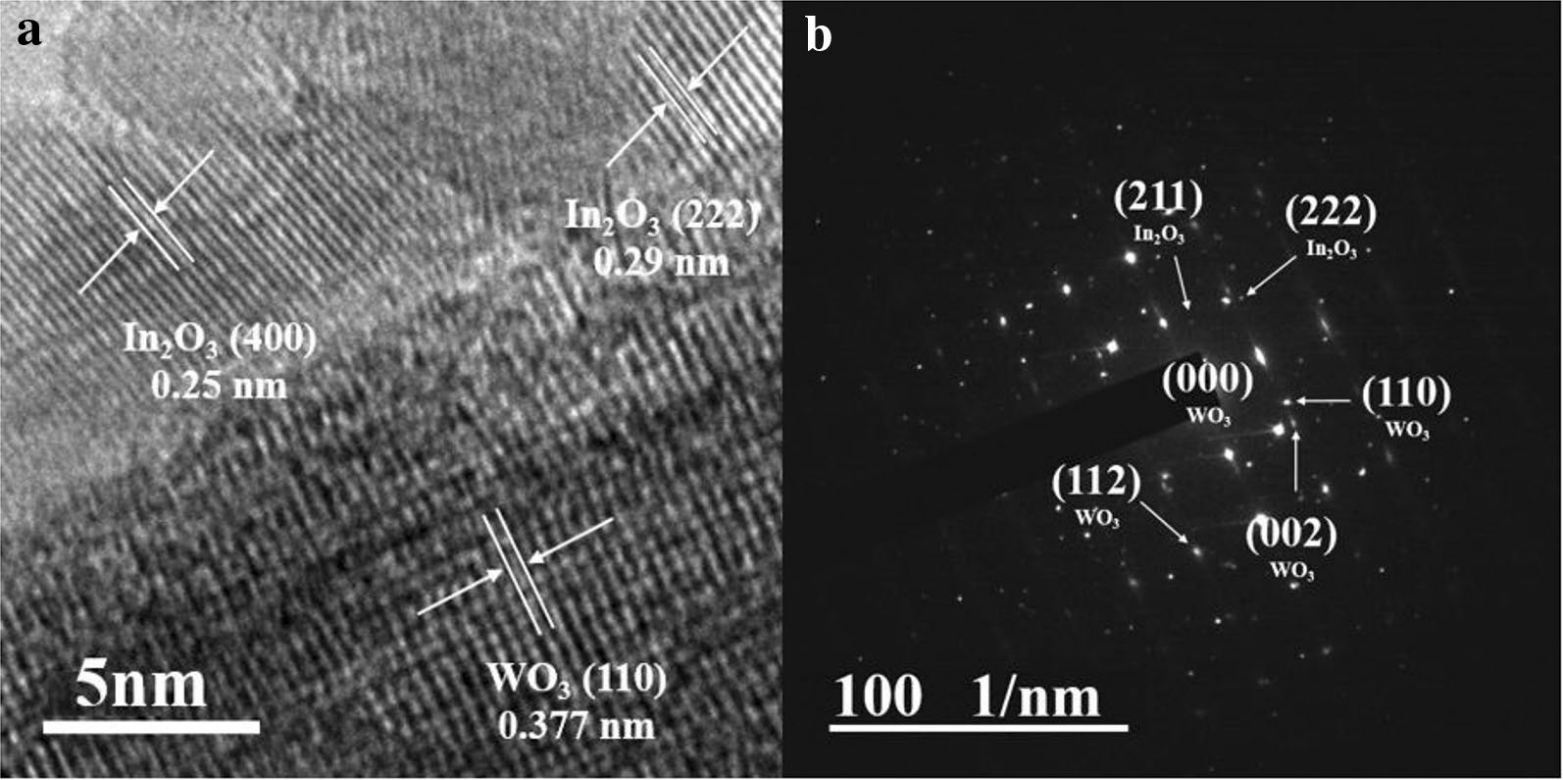
**a** High-resolution TEM image, **b** corresponding electron diffraction pattern of the In_2_O_3_ NPs-decorated WO_3_ NRs

Figure 3a, b present the high-resolution TEM image and corresponding selected area electron diffraction (SAED) pattern of the In_2_O_3_ NP-decorated WO_3_ NRs. The regularly aligned fringes in both the WO_3_ and In_2_O_3_ regions suggest that the WO_3_ and In_2_O_3_ nanostructures are both crystalline. The corresponding spotty electron diffraction (ED) pattern in Fig. 3b reveals that the WO_3_ and In_2_O_3_ nanostructures are single crystals.

**Fig. 4:**
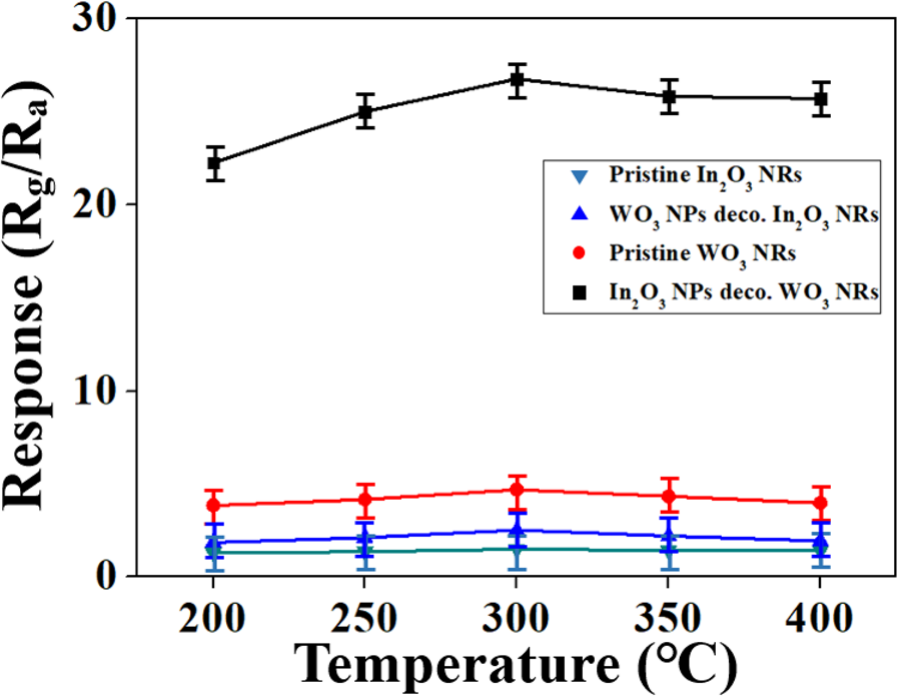
Responses of In_2_O_3_ NPs-decorated WO_3_ NRs and WO_3_ NPs-decorated In_2_O_3_ NPs along with pristine WO_3_ NRs and pristine WO_3_ NRs at various operating temperatures to NO_2_

The temperature-dependent responses of all four different sensor materials to NO_2_ are presented in Fig. 4. The responses of all the four sensor materials to NO_2_ tended to increase with increasing temperature up to 300 °C, and then to decrease with further increases in the temperature. This result suggests that 300 °C is the optimal operating temperature of the sensors in detecting the NO_2_. All the sensing tests hereafter were conducted at 300 °C. At too low operating temperature (250 °C or lower), the NO_2_ molecules may not have enough energy to overcome the energy barrier of adsorption, and fail to be adsorbed on the surface of the sensor materials, WO_3_ and In_2_O_3_. However, at too high operating temperature (350 °C or higher), adsorption failure might also occur because the rate of desorption may outweigh that of adsorption [15].

**Fig. 5:**
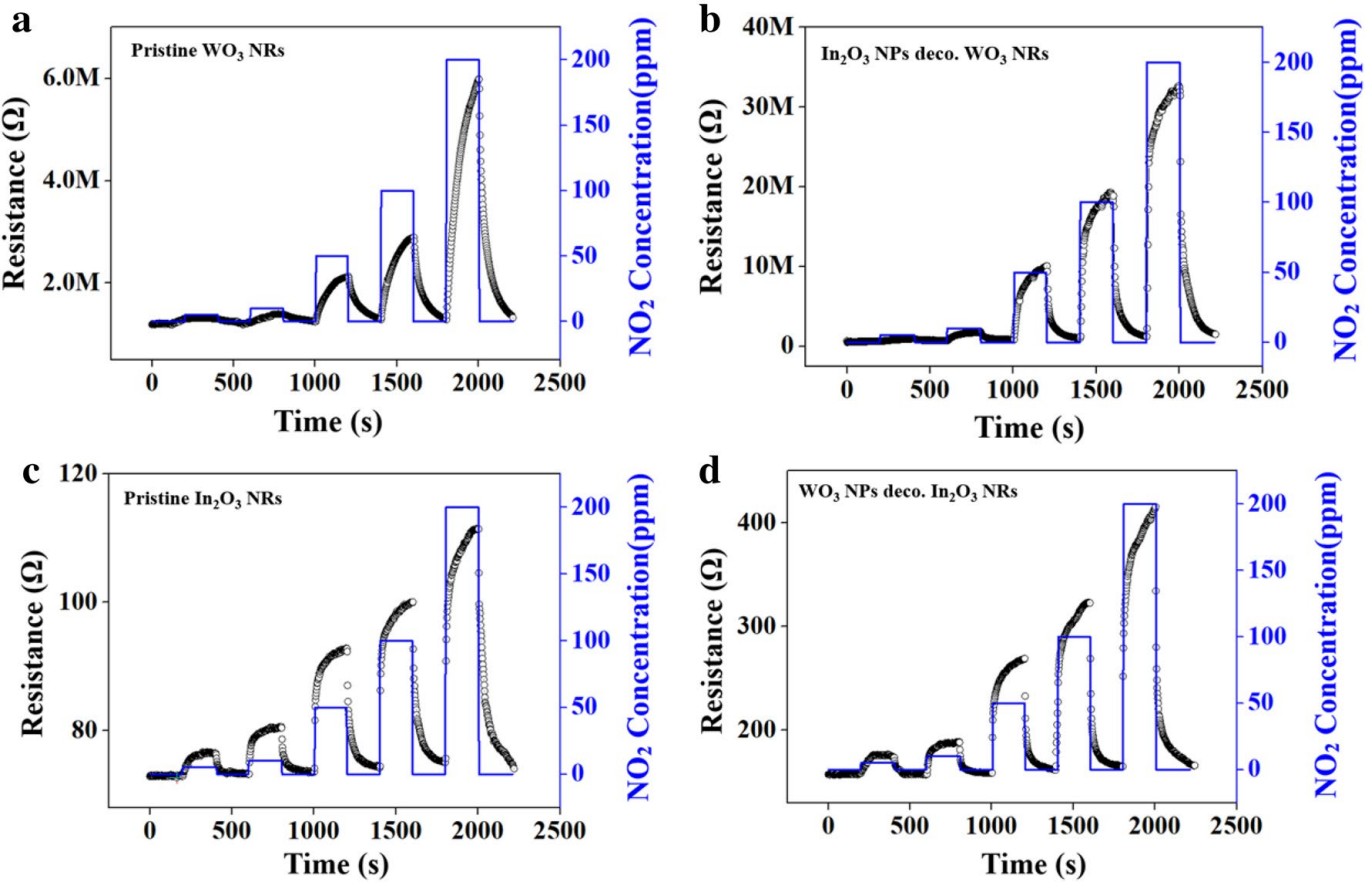
Dynamic response curves: **a** pristine WO_3_ NRs to NO_2_, **b** In_2_O_3_ NPs-decorated WO_3_ NRs to NO_2_, **c** pristine In_2_O_3_ NRs to NO_2_, and **d** WO_3_ NPs-decorated In_2_O_3_ NRs to NO_2_

Figure 5a–d present the dynamic response curves of the four different sensors toward NO_2_. All the sensors showed stable and reversible response and recovery behavior. The resistances of the sensors increased when an oxidizing gas (NO_2_) was supplied, and recovered to the initial value when the NO_2_ supply was stopped and the sensors were exposed to ambient air. This response toward the oxidizing gas is in accord with the sensing behavior of n-type semiconductors. As is well known, both WO_3_ and In_2_O_3_ are n-type semiconductors. The resistance changes increased as the NO_2_ concentration was increased. The starting resistances of the pristine and WO_3_ NP-decorated In_2_O_3_ NRs was markedly lower than the pristine and In_2_O_3_ NPs-decorated WO_3_ NRs, respectively, which might be due to the much lower resistivity of In_2_O_3_ than that of WO_3_.

**Fig. 6:**
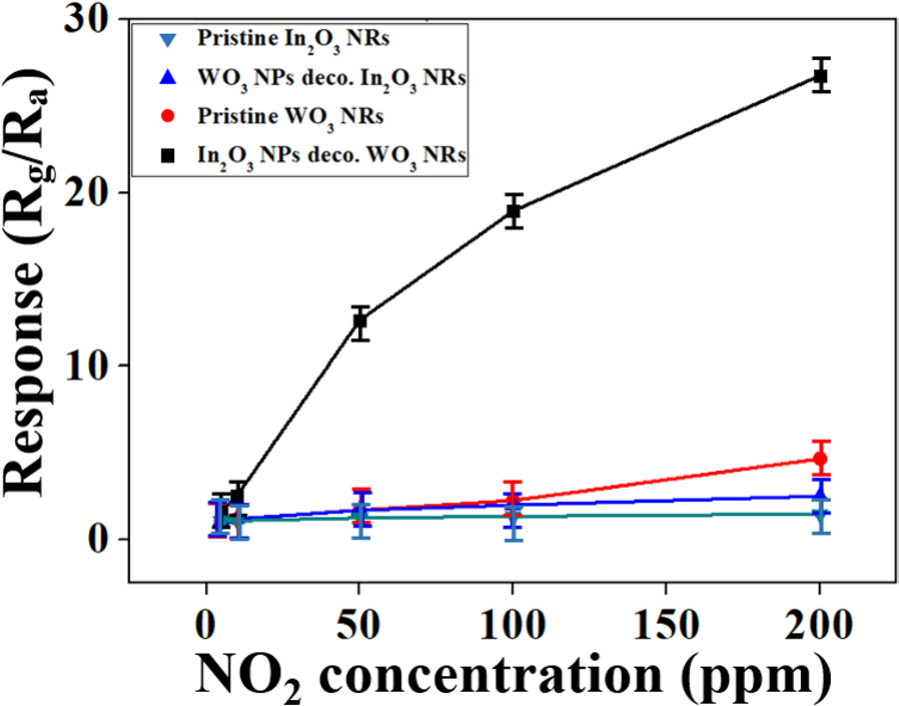
Calibration curves for responses of pristine and In_2_O_3_ NPs-decorated WO_3_ to NO_2_ as well as those of pristine and WO_3_ NPs-decorated In_2_O_3_ NRs to NO_2_ as a function of NO_2_ concentrations

Figure 6 shows the responses of the four different sensors to NO_2_ as a function of the NO_2_ concentration. The response of the In_2_O_3_ NP-decorated WO_3_ NRs to NO_2_ far exceeded those of the other three sensors over the entire NO_2_ concentration range. The more pronounced response of the In_2_O_3_ NP-decorated WO_3_ NR sensor to NO_2_ than that of the pristine WO_3_ NRs and the greater response of the WO_3_ NPs-decorated In_2_O_3_ NRs sensor to NO_2_ than that of the pristine In_2_O_3_ NRs is plausibly due to the resistance modulation at the WO_3_–In_2_O_3_ heterojunction formation [16]. Contrarily, the much stronger response of the In_2_O_3_ NP-decorated WO_3_ NR sensor to NO_2_ than that of the WO_3_ NP-decorated In_2_O_3_ NR sensor is very interesting. The origin of this difference in the response of the heterostructured sensors with inverse configuration is discussed in detail in the next section.

**Fig. 7:**
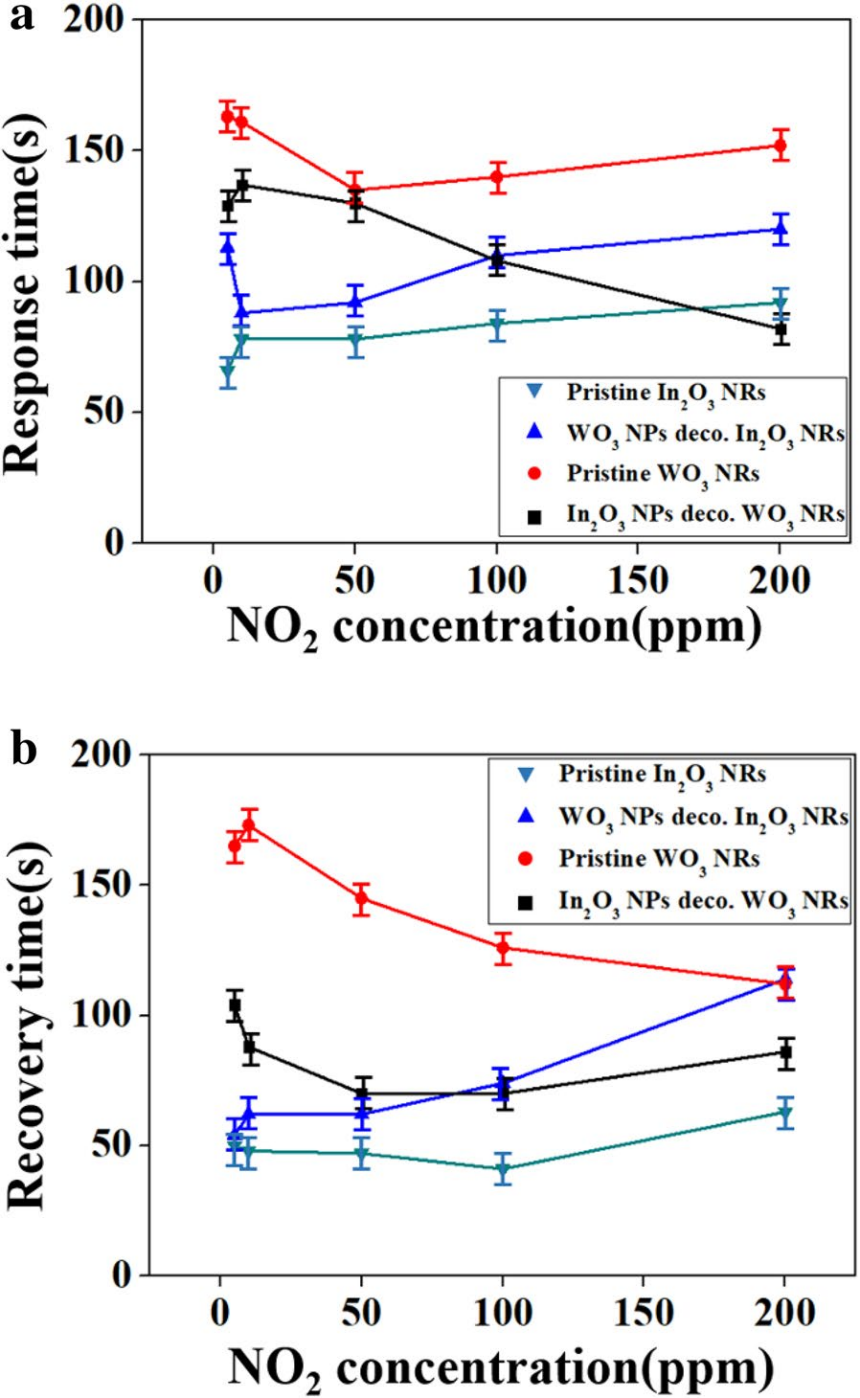
Calibration curves for **a** response times and **b** recovery times of pristine and In_2_O_3_ NPs-decorated WO_3_ NRs as well as pristine and WO_3_ NPs-decorated In_2_O_3_ NRs to NO_2_ as a function of NO_2_ concentrations

Figure 7a, b show the response and recovery times of the four different sensors toward NO_2_ as a function of the NO_2_ concentration. As expected, the response and recovery times of the In_2_O_3_ NP-decorated WO_3_ NR sensor were shorter than those of the pristine WO_3_ NRs. In contrast, the response and recovery times of the WO_3_ NP-decorated In_2_O_3_ NR sensor were longer than those of the pristine In_2_O_3_ NR sensor. Comparison of the response and recovery times of the In_2_O_3_ NP-decorated WO_3_ NR sensor with those of the WO_3_ NP-decorated In_2_O_3_ NR sensor, interestingly, shows shorter response and recovery times for the former in the higher NO_2_ concentration range, whereas longer response and recovery times for the lower NO_2_ concentration range than the latter. Shorter response and recovery times are commonly associated with a higher response for gas sensors.

**Fig. 8:**
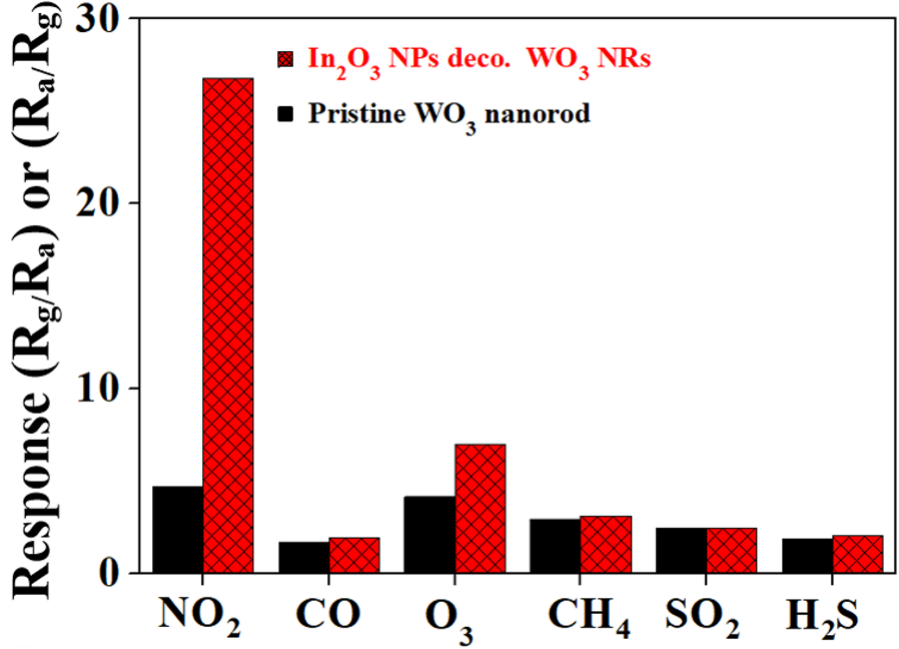
Selectivity patterns of pristine and In_2_O_3_ NPs-decorated WO_3_ NRs toward NO_2_ against other gases such as O_3_, SO_2_, CO, CH_4_, and H_2_S

The response of the In_2_O_3_ NP-decorated WO_3_ NR sensor to various gases is shown in Fig. 8. The sensor showed a much stronger response to NO_2_ than to the other oxidizing gases such as O_3_ and SO_2_ or reducing gases such as CO, CH_4_ and H_2_S, demonstrating the selectivity and sensitivity of the In_2_O_3_ NP-decorated WO_3_ NR sensor toward NO_2_. The selectivity of the sensor toward NO_2_ against other gases might be related to the different optimal operating temperatures of the sensor for different target gases. The response of a sensor material to a certain gas might depend on many factors such as solid solubility of the gas in the material, the decomposition rate of the adsorbed molecule at the material surface, the charge carrier concentration in the material, the Debye length in the material, the catalytic activity of the material, the orbital energy of the gas molecule, etc. The dissociation (or reduction) rate of an oxidizing gas such as NO_2_ is determined by these factors. Therefore, each gas has the characteristic optimal dissociation temperature at which its dissociation rate is maximized. The In_2_O_3_-decorated WO_3_ nanorod sensor fabricated in this study showed higher response fortunately to NO_2_ than other gases at 300 °C because of the higher dissociation rate of NO_2_ at the surface of In_2_O_3_ and WO_3_ at the temperature, but it might show higher responses to other gases than NO_2_ at different temperatures [17–22].

**Fig. 9:**
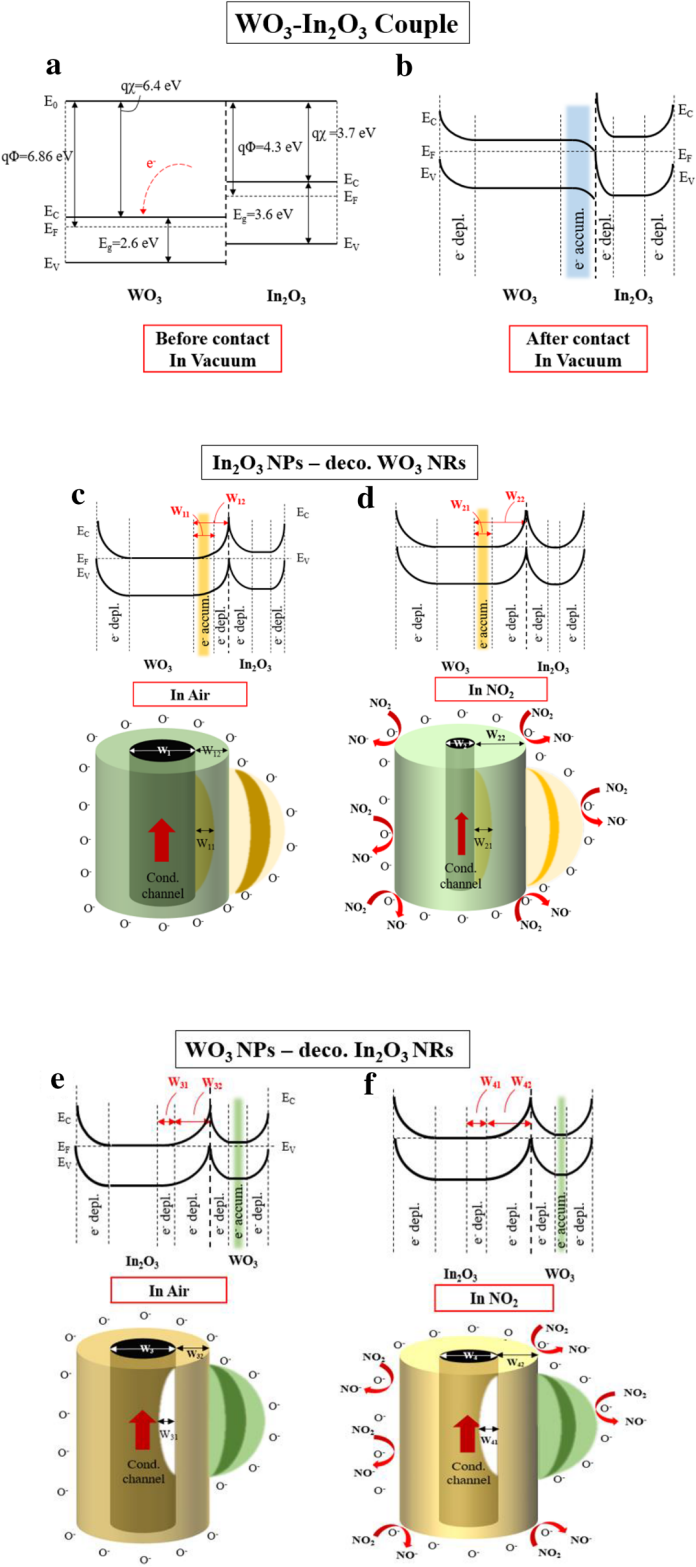
Energy band diagrams of a WO_3_-In_2_O_3_ couple **a** before and **b** after contact. Energy band diagrams and schematics of In_2_O_3_ NPs-decorated WO_3_ NRs: **c** in air and **d** in NO_2_, and of WO_3_ NPs-decorated In_2_O_3_ NRs: **e** in air and **f** in NO_2_

Figure 9a–d illustrate the sensing mechanism of the In_2_O_3_ NP-decorated WO_3_ NR sensor toward NO_2_. Earlier studies reported that the response of a base sensor material could be enhanced by decoration with another type of SMO NPs, mainly because of the greater modulation of the width of the depletion layer or the conduction channel, resulting in the greater modulation of the sensor resistance [23–25]. n-Type WO_3_ has a larger work function (*qΦ*) than n-type In_2_O_3_ (Fig. 9a). Accordingly, if WO_3_ and In_2_O_3_ are in contact, even under vacuum, electron transfer from In_2_O_3_ (with a larger work function) to WO_3_ (with a smaller work function) tends to occur until electronic equilibrium is attained between WO_3_ and In_2_O_3_, as shown in Fig. 9a. Consequently, electron-accumulation and electron-depletion layers are formed in the WO_3_ and In_2_O_3_ regions, respectively. The schematic shows the In_2_O_3_ NP-decorated WO_3_ NRs with an accumulation layer with a width of *W*_*11*_, formed by electron transfer from the In_2_O_3_ NPs to the WO_3_ NRs (Fig. 9b). In ambient air, the surfaces of the WO_3_ NR and In_2_O_3_ NP adsorb oxygen molecules and the adsorbed oxygen molecules are ionized by the capture of the free electrons in the WO_3_ and In_2_O_3_ surface regions (Fig. 9c). Consequently, a depletion layer with a width of *W*_*12*_ is formed in the surface region of WO_3_. The schematic shows a decorated WO_3_ NR with a depletion layer formed via ionization of adsorbed oxygen molecules and an accumulation layer formed by electron transfer from the In_2_O_3_ to the WO_3_ (Fig. 9c). When NO_2_ gas is supplied, NO_2_ and O_2_ molecules are both adsorbed by the In_2_O_3_ and WO_3_ surfaces. The adsorbed NO_2_ molecules are converted into NO_2_^−^ or NO [26, 27] and the adsorbed oxygen molecules are converted into oxygen ions by capturing electrons from the WO_3_ and In_2_O_3_ surface regions. Consequently, a thicker depletion layer (with a width of *W*_*22*_) (Fig. 9d) is formed than that formed in ambient air. The schematic shows a WO_3_ NR with a depletion layer with a width of *W*_*22*_ as well as the accumulation layer with a width of *W*_*21*_ formed by the electron transfer from the In_2_O_3_ to the WO_3_ (Fig. 9d).

The sensing mechanism of the WO_3_ NP-decorated In_2_O_3_ NR sensor toward NO_2_ is illustrated in Fig. 9e, f. As discussed above, electron-accumulation and depletion layers are formed in the WO_3_ and In_2_O_3_ regions, respectively. Thus, a depletion layer with a width of *W*_*31*_ forms on the In_2_O_3_ side of the WO_3_–In_2_O_3_ interface (Fig. 9e). In ambient air, oxygen molecules are adsorbed by the In_2_O_3_ NR surface and ionized by accepting the electrons from the In_2_O_3_ and WO_3_ surface regions. Consequently, a depletion layer with a width of *W*_*32*_ is formed in the In_2_O_3_ surface region. A WO_3_ NP-decorated In_2_O_3_ NR with a depletion layer formed due to the ionization of adsorbed oxygen molecules and a depletion layer formed by electron transfer from the WO_3_ NP is shown in Fig. 9e. Under NO_2_ atmosphere, a thicker depletion layer (with a width of *W*_*42*_) than that generated in ambient air is formed due to the adsorption and ionization of both NO_2_ and O_2_ molecules (Fig. 9f). Note that no electron-accumulation layer is formed in the In_2_O_3_ NR throughout the on–off cycling of the NO_2_ gas supply.

Under ambient air and NO_2_, there was no big difference in the basic response of the In_2_O_3_ NPs-decorated WO_3_ NRs versus that of the WO_3_ NPs-decorated In_2_O_3_ NRs. A relatively thin depletion layer is formed in both samples upon exposure to air and a thick depletion layer is generated upon exposure to NO_2_. Consequently, the width of the conduction channel of the In_2_O_3_ NP-decorated WO_3_ NRs formed upon exposure to NO_2_ is much smaller than that of the WO_3_ NP-decorated In_2_O_3_ NRs formed in ambient air. The conduction channel of the In_2_O_3_ NP-decorated WO_3_ NRs has a room for substantial reduction upon exposure to NO_2_ because the conduction channel width has already been expanded due to the formation of an accumulation layer by the transfer of electrons from the In_2_O_3_ NP to the WO_3_ NR. In contrast, the conduction channel of the WO_3_ NPs-decorated In_2_O_3_ NRs was already shrunken due to the formation of the electron-depletion layer via electron transfer from the In_2_O_3_ NR to the WO_3_ NP. Accordingly, the conduction channel of the In_2_O_3_ NR has little room for further reduction upon exposure to NO_2_ [28].

The response, *S* is defined as *R*_*g*_*/R*_*a*_. for the oxidizing gas NO_2_ and *S* is proportional to *A*_*a*_*/A*_*g*_ because the resistance *R *= *ρl/A*, where *ρ*, *l* and *A* are the density, length and cross-sectional area of the conductor (channel, here) [29]. *S* can be expressed as the ratio of the conduction channel width for an analyte gas to that for air, *S *= *W*_*a*_^*2*^*/W*_*g*_^*2*^ because *A *=* πW*^*2*^, where *W* is the conduction channel width. Therefore, the In_2_O_3_ NP-decorated WO_3_ NR sensor has a higher response, *S* to NO_2_ because of the far smaller conduction channel width, *W*_*g*_ in NO_2_ atmosphere. In contrast, the WO_3_ NP-decorated In_2_O_3_ NR sensor has a lower response, *S* because of the lower contraction of the conduction channel width, *W*_*g*_ in NO_2_ atmosphere.

## Conclusions

The sensing properties of the In_2_O_3_ NP-decorated WO_3_ NR sensor toward NO_2_ were compared to those of the WO_3_ NP-decorated In_2_O_3_ NR sensor. The response of the former sensor to NO_2_ was more pronounced than that of the latter due to the significant reduction of the conduction channel width of the former sensor upon exposure to NO_2_. The conduction channel of the In_2_O_3_ NP-decorated WO_3_ NR sensor had room for sufficient reduction as it was already expanded by electron transfer from the In_2_O_3_ NPs to the WO_3_ NRs. In contrast, the WO_3_ NP-decorated In_2_O_3_ NR sensor showed a lower response due to insufficient reduction of the conduction channel width upon exposure to NO_2_. The conduction channel of the WO_3_ NP-decorated In_2_O_3_ NR sensor had little room for further reduction due to prior shrinkage associated with electron transfer from the In_2_O_3_ NRs to the WO_3_ NPs. For the detection of a reducing gas instead of an oxidizing gas, the magnitude of the sensor response would be reversed. Therefore, choosing a proper decorating material in fabricating n-SMO NR sensors decorated with n-SMO NPs is important in obtaining high sensitivity. An SMO with a smaller work function must be chosen as a decorating material in a decorated heterostructured sensor for oxidizing gas detection. In contrast, an SMO with a larger work function must be chosen as the decorating material for heterostructured sensors geared toward the detection of a reducing gas.

## Data Availability

The datasets used and/or analyzed during the current study are available from the corresponding author on reasonable request.

## Abbreviations

NP: nanoparticle
NR: Nanorod
SMO: semiconducting metal oxide
UV: ultra violet
JCPDS: Joint Committee on Powder Diffraction Standards

## References

[1] Romain AC, Nicolas J (2010). Long term stability of metal oxide-based gas sensors for e-nose environmental applications: an overview. Sens. Actuators B Chem..

[2] Lu G, Ocola LE (2009). Room-temperature gas sensing based on electron transfer between discrete tin oxide nanocrystals and multiwalled carbon nanotubes. Adv. Mater..

[3] Miller DR, Akbar SA, Morris PA (2014). Nanoscale metal oxide-based heterojunctions for gas sensing: a review. Sens. Actuators B.

[4] Dhawale DS, Salunkhe RR, Fulari VJ, Rath MC, Sawant SN, Lokhande CD (2009). Liquefied petroleum gas (LPG) sensing performance of electron beam irradiated chemically deposited TiO_2_ thin films. Sens. Actuators B.

[5] Kim HJ, Lee JH (2014). Highly sensitive and selective gas sensors using p-type semiconductors: Overview. Sens. Actuators B.

[6] Shaposhnik D, Pavelko R, Llobet E, Gispert-Guirado F, Vilano X (2011). Hydrogen sensors on the basis of SnO_2_–TiO_2_ systems. Proc. Eng..

[7] Vasiliev R, Rumyantseva M (1999). Effect of interdiffusion on electrical and gas sensor properties of CuO/SnO_2_ heterostructure. Mater. Sci..

[8] Park S, Kim S, Kheel H, Lee C (2015). Oxidizing gas sensing properties of the n-ZnO/p-Co_3_O_4_ composite nanoparticle network sensor. Sens. Actuators B Chem.

[9] Shaislamov U, Yang BL (2012). CdS sensitized single-crystalline TiO_2_ nanorods and polycrystalline nanotubes for solar hydrogen generation. J. Mater. Res..

[10] Elouali S, Bloor LG, Binions R, Parkin IP, Carmalt CJ, Darr JA (2012). Gas sensing with nano-indium oxides (In_2_O_3_) prepared via continuous hydrothermal flow synthesis. Langmuir.

[11] Yan Y, Chou L (2008). Competitive growth of In_2_O_3_ nanorods with rectangular cross sections. Appl. Phys. A.

[12] Lee JK, Lee WS, Lee WI, Choi SB, Hyun SK, Lee CM (2018). Selective detection of a reducing gas using WO_3_-decorated ZnO nanorod-based sensor in the presence of oxidizing gases. Phys. Status Solidi A.

[13] Shibuya M, Miyauchi M (2009). Site-selective deposition of metal nanoparticles on aligned WO_3_ nanotrees for super-hydrophilic thin films. Adv. Mater..

[14] Kim SH, Park SH, Park SY, Lee CM (2015). Acetone sensing of Au and Pd-decorated WO_3_ nanorod sensors. Sens. Actuators B.

[15] Sun GJ, Lee JK, Choi SB, Lee WI, Kim HW, Lee CM (2017). Selective oxidizing gas sensing and dominant sensing mechanism of n-CaO-Decorated n-ZnO nanorod sensors. ACS Appl. Mater. Interfaces..

[16] Wang W, Li Z, Zheng W, Huang H, Wang C, Sun J (2010). Cr2O3-sensitized ZnO electrospun nanofibers based on ethanol detectors. sens. Actuators B.

[17] Parrondo J, Santhanam R, Mijangos F, Rambabu B (2010). Electrocatalytic performance of In_2_O_3_-supported Pt/C nanoparticles for ethanol electro-oxidation in direct ethanol fuel cells. Int. J. Electrochem. Sci..

[18] Choi KI, Kim HR, Kim KM, Liu D, Cao G, Lee JH (2010). C_2_H_5_OH sensing characteristics of various Co_3_O_4_ nanostructures prepared by solvothermal reaction. Sens. Actuators B: Chem..

[19] Li Y, Xu J, Chao J, Chen D, Ouyang S, Ye J, Shen G (2011). High-aspect-ratio single-crystalline porous In_2_O_3_ nanobelts with enhanced gas sensing properties. J. Mater. Chem..

[20] Wen Z, Tianmo L (2010). Gas-sensing properties of SnO_2_-TiO_2_-based sensor for volatile organic compound gas and its sensing mechanism. Phys. B.

[21] Feng C, Li W, Li C, Zhu L, Zhang H, Zhang Y, Ruan S, Chen W, Yu L (2012). Highly efficient rapid ethanol sensing based on In_2-x_Ni_x_O_3_ nanofibers. Sens. Actuators B Chem..

[22] Park S, Kim S, Sun G, Lee C (2015). Synthesis, structure, and ethanol gas sensing properties of In_2_O_3_ nanorods decorated with Bi_2_O_3_ nanoparticles. ACS Appl. Mater. Interfaces.

[23] Vanheusden K, Warren WL, Seager CH, Tallant DR, Voigt JA, Gnade BE (1996). J. Appl. Phys..

[24] Woo HS, Na CW, Kim ID, Lee JH (2012). Highly sensitive and selective trimethylamine sensor using one-dimensional ZnO–Cr_2_O_3_ heterostructures. Nanotechnology.

[25] Mashock M, Yu K, Cui S, Mao S, Lu G, Chen J (2012). Modulating gas sensing properties of CuO nanowires through creation of discete nanosized p–n junctions on their surfaces. ACS Appl. Mater. Interfaces.

[26] Belysheva TV, Bogovtseva LP, Kazachkov EA, Serebryakova NV (2003). Gas-sensing properties of doped In_2_O_3_ films as sensors for NO_2_ in air. J. Anal. Chem..

[27] Ferro R, Rodríguez JA, Bertrand P (2008). Peculiarities of nitrogen dioxide detection with sprayed undoped and indium-doped zinc oxide thin films. Thin Solid Films.

[28] Choi SW, Katochi A, Kim JH, Kim SS (2015). Striking sensing improvement of n-type oxide nanowires by electronic sensitization based on work function difference. J. Mater. Chem. C.

[29] E. S. Yang, Fundametals of Semiconductor Device (McGraw-Hill Inc., New York, 1978). ISBN 10: 0070722366 ISBN 13: 9780070722361

